# Supplementary figures and images for: Tensile Overload Injures Human Alveolar Epithelial Cells through YAP/F-Actin/MAPK Signaling
Source: Biomedicines. 2023 Jun 26;11(7):1833. doi: 10.3390/biomedicines11071833 (PMC10376431; doi:10.3390/biomedicines11071833)

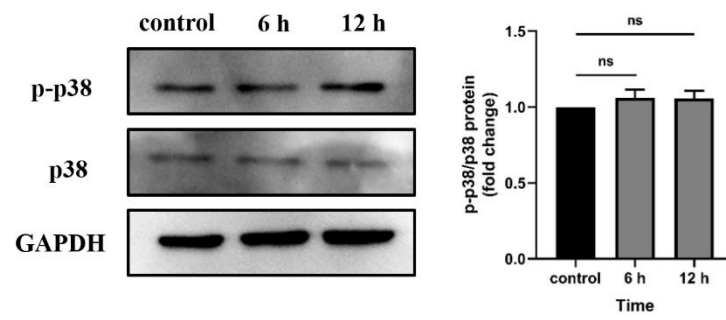

**Figure S1.** Tensile overload on p38 signal pathways in BEAS-2B cells and statistical results.

Supplement: Supplementary file 1 [file biomedicines-11-01833-s001.zip › biomedicines-2423819-supplementary.pdf]
